# miR-96, miR-145 and miR-9 expression increases, and *IGF-1R* and *FOXO1* expression decreases in peripheral blood mononuclear cells of aging humans

**DOI:** 10.1186/s12877-016-0379-y

**Published:** 2016-11-30

**Authors:** Monika Budzinska, Magdalena Owczarz, Eliza Pawlik-Pachucka, Malgorzata Roszkowska-Gancarz, Przemyslaw Slusarczyk, Monika Puzianowska-Kuznicka

**Affiliations:** 1 Department of Geriatrics and Gerontology, Medical Centre of Postgraduate Education, Marymoncka 99/103, 01-813 Warsaw, Poland; 2 Department of Human Epigenetics, Mossakowski Medical Research Centre, PAS, Pawinskiego 5, 02-106 Warsaw, Poland; 3 PolSenior Project, International Institute of Molecular and Cell Biology, Trojdena 4, 02-109 Warsaw, Poland

**Keywords:** Epigenetic drift, Forkhead box O1 transcription factor, Forkhead box O3a transcription factor, Insulin-like growth factor-1 receptor, miRNA, Peripheral blood mononuclear cells, Successful aging

## Abstract

**Background:**

In mammals, the IGF-1 pathway affects the phenotype of aging. Since the function of the immune system is modulated by IGF-1, it is plausible that immunosenescence might in part result from altered control by this pathway. We therefore examined whether the expression of *IGF-1R*, *FOXO1*, and *FOXO3a* in peripheral blood mononuclear cells (PBMC) changes with age and if this might be due to changes in the expression of select miRNAs.

**Methods:**

The expression of *IGF-1R*, *FOXO1*, *FOXO3a*, as well as of miR-9, miR-96, miR-99a, miR-132, miR-145, and miR-182 was examined in PBMC of young (27.8 ± 3.7 years), elderly (65.6 ± 3.4 years), and long-lived (94.0 ± 3.7 years) Polish Caucasians using real-time PCR. mRNA/miRNA interactions were studied in HEK 293 cells using luciferase-expressing pmirGLO reporter vector.

**Results:**

The median expression of *IGF-1R* decreased with age (*p* < 0.000001), as did the expression of *FOXO1* (*p* < 0.000001), while the expression of *FOXO3a* remained stable. We also found an age-associated increase of the median expression of miR-96 (*p* = 0.002), miR-145 (*p* = 0.024) and miR-9 (*p* = 0.026), decrease of the expression of miR-99a (*p* = 0.037), and no changes regarding miR-132 and miR-182. Functional studies revealed that miR-96 and miR-182 interacted with human *IGF-1R* mRNA, and that miR-145 and miR-132 interacted with human *FOXO1* mRNA.

**Conclusions:**

The age-associated higher expression of miR-96 and miR-145 might contribute to the lower expression of *IGF-1R* while the higher expression of miR-96, miR-145 and miR-9 might contribute to the lower expression of *FOXO1* in peripheral blood mononuclear cells of aging humans. Sustained expression/function of *FOXO3a* but not of the other two genes might be important for the maintenance of the immune system function in these individuals.

## Background

Aging is a multifactorial process affecting all tissues and organs, which depends on genetic, epigenetic, environmental and stochastic factors. On the molecular level, one of its key features is aging-associated change of gene activity, to a large extent driven by epigenetic drift, a subtle but progressive change of the epigenome, characterized by global DNA demethylation, hypermethylation of select promoters, histone code alterations and change in miRNAs expression [1–3]. miRNAs are short, non-coding, single-stranded RNA molecules that regulate gene expression at the pre-translational level [4]. Notably, aging-associated change of miRNA expression and its phenotypic effects are not completely elucidated, mostly due to the complex nature of miRNA action and to the fact that miRNA expression changes seems to be, at least in part, tissue-specific [5–7].

The first indications of an involvement of the insulin/IGF-1 pathway in the regulation of lifespan originated from experiments performed on *Caenorhabditis elegans*, in which mutations reducing the activity of daf-2, an ortholog of mammalian IGF-1 and insulin receptors (IGF-1R and IR, respectively), significantly extended the lifespan of this nematode [8]. The effect has been shown to be mediated by daf-16, an ortholog of mammalian forkhead transcription factors (FOXO). Stimulation of IGF-1R leads to Akt-dependent phosphorylation of FOXO, its sequestration in the cytoplasm, and to inhibition of its longevity-promoting activity [9, 10],while overexpression of FOXO increases the duration of life [11, 12]. Involvement of this pathway in longevity control was further confirmed in *Drosophila melanogaster* [13, 14]. In mice, heterozygous deletion of *IGF-1R* (null animals were not viable) resulted in lifespan extension by 33% in females [15], however, this was not replicated by other authors [16]. In humans, genetic variants of *FOXO3A* were quite consistently reported as associated with longevity [17–19]. In contrast, only a few scientific reports described an association of the *IGF-1R* and *FOXO1* variants with longevity [18, 20], while others did not see such associations [21, 22]. Therefore, the effect of IGF-1 pathway on longevity in mammals in general and in humans in particular, remains a controversial issue. Nonetheless, it is well established that modifications of this pathway significantly affect the phenotype of aging, as age-related decrease in circulating IGF-1 levels contributes to the development of cardiovascular disease, stroke, type 2 diabetes mellitus, osteoporosis, sarcopenia, and frailty but, at the same time, lowers the risk of cancer [23–28].

An integral part of aging is immunosenescence. Its most important features are: a decreased number of naïve T and B lymphocytes, an increased number of memory and effector T and B lymphocytes as well as of natural killer cells the function of which is altered, an impaired production of interleukins and cytokines, an overproduction of autoantibodies and production of less effective antibodies, all leading to a low-grade chronic inflammation, a decreased response to pathogens and to immunization, and to the increased risk of autoimmunity and cancers [29]. The function of the immune system depends on numerous factors and pathways, including the growth hormone/IGF-1 pathway [30, 31], the function of which also varies with age, being one of the hypothetical causes of immunosenescence. Indeed, a significant correlation has been demonstrated between plasma IGF-1 levels and the T lymphocyte (but not B-lymphocyte) proliferative response in young and elderly humans [32]. Furthermore, in aged female rhesus monkeys, administration of recombinant human IGF-1 resulted in an increased percentage of circulating B lymphocytes and of CD8 cells as well as of antibody production to tetanus toxoid [33].

The biological effects of IGF-1 depend not only on the concentration of this factor, but also on the expression and activity of its receptor and of other proteins, that form the IGF-1 pathway. Therefore, the main objective of our work was to establish whether the expression of major components of the IGF-1 axis, *IGF-1R*, *FOXO1* and *FOXO3a*, changes with age in human peripheral blood mononuclear cells (PBMC). Our second objective was to establish whether an altered expression of the selected miRNAs might contribute to *IGF-1R*, *FOXO1* and *FOXO3a* age-associated expression differences.

## Methods

### Study subjects

Polish Caucasians were divided into the young (Y, *n* = 56, age range 20–39 years, mean age 27.8 ± 3.7 years, 29 females, 27 males), elderly (E, *n* = 52, 60–73 years, 65.4 ± 3.4 years, 25 females, 27 males), and long-lived (L, *n* = 48, 90–102 years, 94.2 ± 3.7 years, 31 females, 17 males) age groups (Table 1). They were non-obese, without signs and symptoms of current infection, and without history of myocardial infarction, stroke, type 2 diabetes mellitus, cancer, or neurodegeneration. However, in the E and L groups moderate hypertension was allowed, and some study participants belonging to the L group had a mild degree physical or cognitive disability. Physical performance and cognitive functioning were assessed during recruitment for the study with the Activities of Daily Living (ADL) scale [34] and Mini-Mental State Examination (MMSE) [35], respectively. The following cut-offs were used for physical performance: ADL score 5–6 – independent, 3–4 – partially dependent, 0–2 – totally dependent, and for cognitive functioning: MMSE score 28–30 – normal cognition, 24–27 – minimal cognitive impairment, 20–23 – mild, 10–19 – moderate, <10 – severe cognitive impairment. Fifteen percent of elderly and 41% of long-lived individuals were taking low-dose acetylsalicylic acid. All participants gave a written informed consent for participation in the study. The anonymity of patients has been preserved at all stages of this investigation. The study protocol was approved by the Bioethics Committee of the Medical University of Warsaw.

**Table 1:** Basic clinical and biochemical parameters of the elderly and long-lived study subjects

	Elderly	Long-lived
	*n* = 52	*n* = 48
Age^a^ (years)	65.6 ± 3.4	94.2 ± 3.8
BMI^a^ (kg/m^2^)	28.1 (25.7, 30.10)	25.3 (21.9, 28.6)
Fasting glucose^a^ (mg/dl)	90.2 (81.1, 96.7)	87.5 (83.8, 92.5)
Total cholesterol^a^ (mg/dl)	203.2 (177.1, 239.85)	173 (151.8, 203.9)
HDL^a^ (mg/dl)	56 (49.5, 62.3)	51.9 (47, 67.1)
IL-6^a^ (pg/ml)	1.5 (0.8, 2.8)	2.9 (1.4, 4.6)
CRP^a^ (mg/l)	1.9 (1.1, 4.1)	2.4 (1.5, 10.7)
MMSE^a^	29 (27, 29)	19 (17, 26)
ADL^a^	6 (6, 6)	6 (4, 6)

^a^ median (25^th^, 75^th^ percentile)

### Isolation of PBMC and isolation of RNA

Human peripheral blood mononuclear cells were isolated as previously described [36]. Next, since the available data indicated that TRIzol (Invitrogen, Carlsbad, CA, USA) is suitable for isolation of both long and short RNAs and their further analysis using RT-PCR, next generation sequencing, etc., and that it is not inferior to other isolation methods [37–41], we used this reagent to isolate total RNA according to the procedure supplied by the manufacturer. The integrity of RNA was assessed using Agilent 2100 Bioanalyzer (Agilent, CA, USA).

### Reverse transcription

To obtain cDNA for analysis of gene expression, the reactions were performed with 100 ng of PBMC total RNA and with random hexamers using the RevertAid^TM^ H Minus First Strand cDNA Synthesis Kit (Thermo Scientific, Vilnius, Lithuania). For analysis of miRNA expression, reverse transcriptions were performed with 100 ng of total RNA and with a poly-T primer with a 3’ degenerate anchor and a 5’ universal tag from the miRCURY LNA™ Universal RT microRNA PCR system kit (EXIQON, Vedbaek, Denmark).

### Real-time quantification of gene expression and of miRNA expression

The expression of *IGF-1R, FOXO1* and of *FOXO3* was analyzed with semi-quantitative real-time PCR using the LightCycler 480 SYBR Green I Master kit (Roche Diagnostic, Mannheim, Germany) in the Light Cycler 480 (Roche Diagnostic, Mannheim, Germany). The primers for *IGF-1R* were: forward 5’TGAAAGTGACGTCCTGCATTTC3’ and reverse 5’GGTACCGGTGCCAGGTTATG3’, for *FOXO1*: forward 5’TGGACATGCTCAGCAGACATC3’ and reverse 5’TTGGGTCAGGCGGTTCA3’, and for *FOXO3a*: forward 5’GAACGTGGGGAACTTCACTGGTGCTA3’ and reverse 5’GGTCTGCTTTGCCCACTTCCCCTT3’. The reaction was carried out as follows: 5 min at 95 °C, 45 cycles of 12 s at 95 °C, 12 s at 60 °C and 12 s at 72 °C, followed by a melting curve cycle. The results were normalized against the expression of the *ACTB* gene. Each reaction was performed in duplicate.

To evaluate the expression of miRNAs, a real-time PCR was performed with the miRCURY LNA™ Universal RT microRNA PCR system and SYBR Green kits (EXIQON, Vedbaek, Denmark) in the Light Cycler 480, according to the manufacturer’s protocol. The reaction conditions were: 10 min at 95 °C, 50 cycles of 10 s at 95 °C, 1 min at 60 °C, followed by melting curve cycle. The results were normalized against the expression of endogenous control U6 snRNA. Each reaction was performed in duplicate.

### Functional analysis of miRNA

Candidate miRNAs were searched for using *in silico* analysis with the TargetScanHuman [42], miRanda-mirSVR [43] and the Pictar [44] programs. Using this approach, we selected miR-96, miR-99a, miR-145, and miR-182 for *IGF-1R* mRNA, and miR-9, miR-96, miR-132, miR-145, and miR-182 for *FOXO1* mRNA.

DNA corresponding to the 5’ end (721 bp) of 3’UTR of *IGF-1R* mRNA was amplified with Dream Taq polymerase (Thero Scientific, Vilnius, Lithuania) with forward 5’ACTA**GAGCTC**GACCTGCTGATCCTTGG3’ (added *Sac*I restriction site shown in bold, the STOP codon is underlined) and reverse 5’TAAG**CTCGAG**AGCTGTCTCTCAAATGG3’ (additional *Xho*I restriction site shown in bold) primers. The PCR reaction conditions were: 4 min at 94 °C, 5 cycles of 1 min at 94 °C, 1 min at 56 °C, 3 min at 72 °C, 35 cycles of 1 min at 94 °C, 1 min at 60 °C and 3 min at 72 °C, and final extension for 10 min at 72 °C. The PCR product was cloned into the pmirGLO reporter vector (Promega, Madison, WI, USA) and sequenced (pmirGLO_IGF-1R_5’ reporter vector). DNA corresponding to the 3’ end (1327 bp) of 3’UTR of *IGF-1R* mRNA was cloned with forward 5’ACTA**GAGCTC**CACTGAGGCACATCATGG3’ (added *Sac*I site is shown in bold) and reverse 5’TAAG**CTCGAG**AGTGATCGTTATGTTCTCGC3’ (added *Xho*I site is shown in bold) primers. The PCR conditions and cloning (pmirGLO_IGF-1R_3’ reporter vector) were the same as above.

DNA corresponding to the 5’ end (1201 bp) of 3’UTR of *FOXO1* mRNA was cloned using forward 5’ACTA**GAGCTC**TGTCAGGCTGAGGGTTAG3’ (added *Sac*I site is shown in bold, the STOP codon is underlined) and reverse 5’CTAA**CTCGAG**CTTGATGCTATGCAGTACG3’ (added *Xho*I site is shown in bold) primers, while for cloning of the 3’ end of this mRNA (1358 bp) the starters were forward 5’ACTA**GAGCTC**CTCTATCATCCTCATTTTGG3’ (added *Sac*I site is shown in bold) and reverse 5’TAAG**CTCGAG**GGCTGACAAGACTTAACTC3’ (added *Xho*I site is shown in bold). Both fragments were amplified under the following PCR conditions: 4 min at 94 °C, 5 cycles of 1 min at 94 °C, 1 min at 56 °C, 3 min at 72 °C, 35 cycles of 1 min at 94 °C, 1 min at 58 °C and 3 min at 72 °C, and final extension for 10 min at 72 °C, and then cloned (pmirGLO_FOXO1_5’ and pmirGLO_FOXO1_3’ reporter vectors, respectively) and sequenced.

HEK 293 cells were cultured in a 96-well dish in Dulbecco Modified Eagle’s medium (Sigma Aldrich, St. Louis, MO, USA) supplemented with 10% heat-inactivated fetal bovine serum, without antibiotics, in a humidified incubator with 5% CO_2,_ at 37 °C. Cells were transfected at 80% confluency with 0.5 μl lipofectamine 2000 (Invitrogen, Life Technologies, Carlsbad, CA, USA) in 50 μl Opti-MEM I medium (Gibco, Life Technologies, Grand Island, NY, USA) without serum, according to the lipofectamine manufacturer’s protocol. Eighty ng of pmirGLO with or without cloned 3’UTR-encoding DNA and 5 pmol of pre-miRNA (pre-miR-96, pre-miR-182 or pre-miR miRNA Precursor Negative Control #2 for *IGF-1R*, and pre-miR-145, pre-miR-132 or pre-miR miRNA Precursor Negative Control #2 for *FOXO1*, Ambion, Life Technologies, Carlsbad, CA USA) were used. Cells were then cultured for 24 h without changing medium, washed with phosphate-buffered saline, and lysed for 15 min with 20 μl Passive Lysis Buffer (Promega, Madison, WI, USA) on a rocking platform. The luminescence was assessed in the Centro XS^3^ LB 960 luminometer (Berthold Technologies, Bad Wilbad, Germany). The luminescence of firefly luciferase substrate was normalized against that of *Renilla* luciferase substrate. Each experiment was repeated 15 times.

### Statistical analysis

Statistical calculations were performed using STATISTICA 10. To assess normality of the distribution, the Shapiro-Wilk test was used. Since the distribution of the expressions of the genes of interest and of miRNAs was not normal, statistical analyses were performed with the Kruskal-Wallis test. The effect of low-dose acetylsalicylic acid on gene and miRNA expression was analyzed with U Mann–Whitney test. The effect of miRNA interaction with the respective mRNA on the reporter protein activity was analyzed by the two-sided Student’s t test. Correlation between the mRNA and the studied miRNA expressions was calculated by the Spearman’s rank correlation coefficient. For all tests the level of significance was established at 0.05.

## Results

### Expression of the IGF-1R, FOXO1, and FOXO3a mRNA in PBMC of young, elderly, and long-lived individuals

We first established whether age affected the mean Cp values for the *ACTB* control gene. We found that they did not differ between age groups (Y: 20.4 ± 0.8, E: 21 ± 1.9, L: 20.1 ± 0.8) and concluded that *ACTB* can be used as the internal control in assessing the expression of genes of interest. The median (25^th^, 75^th^ percentile) expression of *IGF-1R* expressed in arbitrary units was not statistically different in men and women or in low-dose acetylsalicylic acid users and non-users, and further analyses were performed for all study subjects together. The median expression of *IGF-1R* was 1.04 (0.85, 1.34), 0.62 (0.48, 0.91), and 0.57 (0.43, 0.74) in the Y, E, and L groups, respectively, and significantly decreased with age (*p* < 0.00001). The differences between the Y and E, as well as Y and L groups were significant (*p* = 0.000006 and *p* < 0.000001, respectively), while the difference between the E and L groups was not.

The median expression of *FOXO1* expressed in arbitrary units was similar in men and in women, as well as in low-dose acetylsalicylic acid users and non-users, and all study subjects were pooled for further analyses. The median expression of this gene was 1.02 (0.8, 1.37), 0.78 (0.49, 1.12), and 0.61 (0.47, 0.78) in the Y, E, and L groups, respectively, and significantly decreased with age (*p* < 0.000001). The differences were significant for the Y *vs.* E and Y *vs.* L groups (*p* = 0.0013 and *p* < 0.000001, respectively), while for the E *vs*. L groups it was not significant.

Finally, the median expression of *FOXO3a* was not associated with gender nor with low-dose acetylsalicylic acid use. Its median expression was 0.95 (0.79, 1.17), 0.87 (0.66, 1.14), and 1.0 (0.77, 1.2) in the Y, E, and L groups, respectively, and was not dependent on age.

### Interaction of miRNAs with the IGF-1R mRNA and FOXO1 mRNA

Since the interaction between the *IGF-1R* mRNA *vs*. miR-99a or miR-145, as well as the *FOXO1* mRNA *vs*. miR-9, miR-96, or miR-182 has been previously shown by other authors [45–48], we conducted functional studies only for the *IGF-1R* mRNA *vs*. miR-96 and miR-182, and for the *FOXO1* mRNA *vs.* miR-145 and miR-132.

**Fig. 1:**
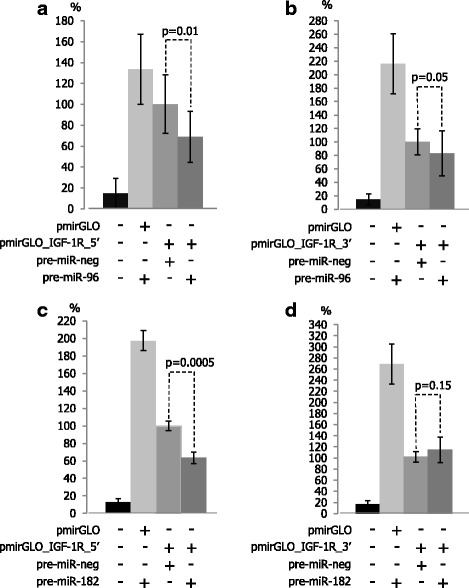
miR-96 and miR-182 interact with the 3’UTR of *IGF-1R* mRNA. HEK 293 cells were co-transfected with the pmirGLO reporter vector and miRNA precursors. The mean relative luminescence induced by firefly luciferase expressed from the reporter vectors containing cloned DNA corresponding to the 3’UTR fragments, in the presence of negative control miRNA, was normalized to 100%. miR-96 interacts with two (**a**, **b**), and miR-182 with one out of two *in silico* indicated binding sites (**c**, **d**). pmirGLO: “empty” reporter vector; pmirGLO_IGF-1R_5’: reporter vector containing DNA corresponding to the 5’ end of 3’UTR of IGF-1R mRNA; pmirGLO_IGF-1R_3’: reporter vector containing DNA corresponding to the 3’ end of 3’UTR of IGF-1R mRNA; pre-miR-neg, pre-miR-96, pre-miR-182: miRNA precursors

3’UTR of *IGF-1R* mRNA contains two putative miR-96 and two putative miR-182 binding sites; therefore, each site was analyzed separately. The mean luminescence induced by firefly luciferase expressed from the reporter vectors pmirGLO_IGF-1R_5’ or pmirGLO_IGF-1R_3’ in the presence of negative control miRNA was normalized to 100%. Co-transfection of pmirGLO_IGF-1R_5’ or pmirGLO_IGF-1R_3’ with pre-miR-96 (Fig. 1a, b) decreased luminescence on average by 31% (*p* = 0.01) and 17.2% (*p* = 0.05), respectively, showing that miR-96 interacts with both fragments of the 3’UTR of *IGF-1R* mRNA and decreases translation of the reporter protein. Co-transfection with pre-miR-182 showed that only the binding site located within the 5’ fragment of 3’UTR of *IGF-1R* mRNA (Fig. 1c) was functional since the luminescence induced by firefly luciferase expressed from the pmirGLO_IGF-1R_5’ vector decreased by 29.5% (*p* = 0.0005). In contrast, there was no interaction between miR-182 and its second putative binding site (Fig. 1d).

**Fig. 2:**
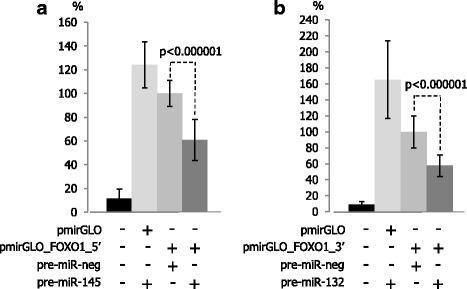
miR-145 and miR-132 interact with the 3’UTR of *FOXO1* mRNA. HEK 293 cells were co-transfected with the pmirGLO reporter vector and miRNA precursors. The mean relative luminescence induced by firefly luciferase expressed from the reporter vectors containing cloned 3’UTR fragments in the presence of negative control miRNA was normalized to 100%. Both miR-145 (**a**) and miR-132 (**b**) interact with *in silico* indicated binding sites. pmirGLO: “empty” reporter vector; pmirGLO_FOXO1_5’: reporter vector containing DNA corresponding to the 5’ end of 3’UTR of FOXO1 mRNA; pmirGLO_FOXO1_3’: reporter vector containing DNA corresponding to the 3’ end of 3’UTR of FOXO1 mRNA; pre-miR-neg, pre-miR-145, pre-miR-132: miRNA precursors

Similarly, the mean luminescence induced by firefly luciferase expressed from the reporter vectors pmirGLO_FOXO1_5’ or pmirGLO_FOXO1_3’ in the presence of negative control miRNA was normalized to 100%. Co-transfection of pmirGLO_FOXO1_5’ and pre-miR-145 (Fig. 2a) decreased luminescence by 39.3% (*p* < 0.000001). Co-transfection of pmirGLO_FOXO1_3’ with pre-miR-132 (Fig. 2b) decreased luminescence by 42.4% (*p* < 0.000001) suggesting that *in silico-*designated single binding sites for these miRNAs were functional.

### Age-dependent changes of expression of miRNAs in PBMC

The median expression of any of the tested miRNAs did not differ between men and women, or between low-dose acetylsalicylic acid users and non-users, and all study subjects were analyzed together. The median expression of miR-96 increased with age (*p* = 0.002 for the whole tested group, Y *vs.* E: *p* = 0.009, Y *vs.* L: *p* = 0.006), as did the median expression of miR-145 (*p* = 0.024 for the whole group, E *vs*. L: *p* = 0.029) and of miR-9 (*p* = 0.026 for the whole group, Y *vs*. L: *p* = 0.021). In contrast, the expression of miR-99a decreased with age (*p* = 0.037 for the whole group, Y *vs.* E: *p* = 0.038). The expression of miR-132 and miR-182 remained stable (Table 2).

**Table 2:** Age-related changes of the expression of the selected miRNAs in human peripheral blood mononuclear cells

	Expression arbitrary units [median value (25^th^, 75^th^ percentile)]			*P*-value		
miRNA	Y^a^	E^b^	L^c^	Y vs. E	E vs. L	Y vs. L
miR-9	0.39 (0.16, 0.82)	0.54 (0.16, 1.13)	0.96 (0.27, 1.40)	*p* = 766	*p* = 0.381	*p* = 0.021
miR-96	0.29 (0.10, 0.53)	0.75 (0.22, 2.06)	0.74 (0.23, 2.52)	***p =*** **0.009**	*p* = 1	***p =*** **0.006**
miR-99a	2.42 (1.16, 4.83)	1.23 (0.53, 2.59)	1.50 (0.83, 3.41)	***p =*** **0.038**	*p* = 1	*p* = 0.265
miR-132	0.21 (0.08, 0.41)	0.21 (0.11, 0.41)	0.25 (0.12, 0.50)	*p* = 1	*p* = 1	*p* = 1
miR-145	0.14 (0.07, 0.47)	0.13 (0.06, 0.34)	0.27 (0.13, 0.57)	*p* = 1	***p =*** **0.029**	*p* = 0.104
miR-182	0.18 (0.10, 0.47)	0.21 (0.06, 1.02)	0.23 (0.09, 1.55)	*p* = 1	*p* = 0.752	*p* = 0.292

^a^: young (Y), ^b^: elderly (E), ^c^ long-lived (L) individuals

Significant *p*-values are shown in bold

There was a weak, but significant negative correlation between the expression of *FOXO1* and expression of miR-96 (Rs = −0.202, *p* = 0.017).

## Discussion

In this work we showed that the expression of key components of the IGF-1 pathway, the *IGF1-R* and *FOXO1* genes, decreases with age, while the median expression of *FOXO3a* remains stable in PBMC of aging humans. The increased function of the IGF-1 pathway has been shown to enhance proliferation of T lymphocytes, promote their survival and stimulate the production of TNF-α and IL-8, stimulate B lymphocyte proliferation and differentiation and enhance immunoglobulin production, as well as to enhance natural killer cell activity, thus exerting a pro-inflammatory effect [49–52]. On the other hand, by stimulation of IL-10 expression and inhibition of Th-1-mediated immune responses in activated T lymphocytes, the increased function of this pathway exerts anti-inflammatory actions [53]. Upregulation of FOXO1 increases the number of naive T and B lymphocytes, accompanies T and cell maturation and supports their homing to lymph nodes [54–56]. It maintains their quiescence, and contributes to immune tolerance [55, 57].

Therefore, an age-associated decrease of expression of *IGF-1R* and *FOXO1* in these cells likely contributes to altered antibody production and natural killer function, as well as to alterations and imbalance in the production of pro-inflammatory and anti-inflammatory agents termed inflammaging, a phenomenon increasing the risk of developing aging-related diseases [58, 59]. Notably, the effects of the downregulation of *IGF-1R* and *FOXO1* might be additive on some immune functions but opposite on others.

It should be noted, however, that the decrease of *IGF-1R* and *FOXO1* expression was also observed in PBMC of long-lived individuals who had never been diagnosed with any aging-related disease. This suggests that in these individuals most possibly genetically predisposed to longevity, such a decrease either is not very harmful to the immune system, or is compensated by other factors. For example, the effect of overproduction of pro-inflammatory agents can be overcome by the increased production of anti-inflammatory factors (anti-inflammaging) [60, 61]. Nevertheless, it is highly relevant to establish whether downregulation of these genes in PBMC of persons not predisposed to longevity or subjected to the negative influences of environmental factors is also of minor importance or, on the contrary, contributes to their increased morbidity and mortality.

Remarkably, the expression of *FOXO3a* in PBMC of our study participants was not affected by age. FOXO3a has been shown to play a pivotal role in maintaining the hematopoietic stem cell, T cell progenitor, and memory T cell pools, in B cell differentiation and persistence of memory B cells, and in promoting survival of natural killer cells [62]. The fact that our results show its expression to be similar independently of age, suggests that its sustained function might be crucial for the maintenance of the immune system function during aging.

In this work we also attempted to elucidate the mechanisms underlying the observed age-dependent differences in the expression of *IGF-1R* and *FOXO1*. Molecular mechanisms affecting gene/protein expression are multiple and include those that influence the rate of transcription, mRNA maturation, transport and stability, the rate of translation, etc. miRNAs activity seems to be one of the important mechanisms affecting gene expression in the context of aging, because the change of miRNAs expression is one of the features of aging-associated epigenetic drift [6]. We showed that the median expression of miR-96 and miR-145, both functionally interacting with the *IGF-1R* and *FOXO1* mRNAs, as well as of miR-9 interacting with the *FOXO1* mRNA, was higher in PBMC of aging humans than of young study subjects. We therefore propose that such an increase might be among the factors that contribute to the decreased expression of *IGF-1R* and *FOXO1* in these cells. In the case of other tested miRNAs, since their expression did not change with age, we conclude that they do not participate in this phenomenon.

This work has some limitations. We studied unfractionated blood mononuclear cells, conscious that the gene expression changes and some features of epigenetic drift might be cell type-specific. Since the percentage of immune cell subtypes changes with age also in apparently healthy humans, the described changes in expression might in part reflect such a quantitative change. In addition, analysis at the protein level would strengthen the validity of our results. However, even though blood is the only biological material easily obtainable from living human donors, the amount of it that we were allowed to collect from the elderly and long-lived individuals was insufficient to carry out efficient fractionation and subsequent analyses. In addition, fractionation followed by cell culturing could increase the number of cells, but we decided not to do so because this could affect the expression of miRNAs and genes. Another reason for studying changes in PBMC was that aging studies involving living humans are commonly carried out on blood cells and using PBMC allowed the comparison of our results with other data, both published [63–65] and yet to be published.

## Conclusions

To sum up, in this work we showed that aging is associated with a decreased expression of *IGF-1R* and *FOXO1* in human PBMC and that this in part can be the result of epigenetic drift. We also show that the sustained expression of *FOXO3a* might be important for the maintenance of the immune system function in aging humans.

## Abbreviations

3’UTR: 3’ untranslated region of mRNA
*ACTB*: Gene encoding β-actin
BMI: Body mass index
FOXO1: Forkhead box O1 transcription factor
FOXO3a: Forkhead box O3a transcription factor
HEK 293: Human embryonic kidney 293 cells
IL-8, IL-10: Interleukin-8, -10
IGF-1R: Insulin-like growth factor-1 receptor
miRNA: microRNA
PBMC: Peripheral blood mononuclear cells
TNF-α: Tumor necrosis factor α
U6 snRNA: U6 small nuclear RNA
